# Extensive blood transcriptome analysis reveals cellular signaling networks activated by circulating glycocalyx components reflecting vascular injury in COVID-19

**DOI:** 10.3389/fimmu.2023.1129766

**Published:** 2023-01-26

**Authors:** Melanie Borrmann, Florian Brandes, Benedikt Kirchner, Matthias Klein, Jean-Noël Billaud, Marlene Reithmair, Markus Rehm, Gustav Schelling, Michael W. Pfaffl, Agnes S. Meidert

**Affiliations:** ^1^ Department of Anesthesiology, University Hospital, Ludwig-Maximilians-University Munich, Munich, Germany; ^2^ Division of Animal Physiology and Immunology, School of Life Sciences Weihenstephan, Technical University of Munich, Freising, Germany; ^3^ Department of Neurology, University Hospital, Ludwig-Maximilians-University of Munich, Munich, Germany; ^4^ QIAGEN Digital Insights, Redwood City, United States; ^5^ Institute of Human Genetics, University Hospital, Ludwig-Maximilians-University Munich, Munich, Germany; ^6^ Department of Anesthesiology and intensive Care Medicine, Hospital Agatharied, Hausham, Germany

**Keywords:** small RNA, COVID-19, glycocalyx, acute respiratory distress syndrome, endothelial dysfunction, cell-free microRNAs, extracellular vesicles

## Abstract

**Background:**

Degradation of the endothelial protective glycocalyx layer during COVID-19 infection leads to shedding of major glycocalyx components. These circulating proteins and their degradation products may feedback on immune and endothelial cells and activate molecular signaling cascades in COVID-19 associated microvascular injury. To test this hypothesis, we measured plasma glycocalyx components in patients with SARS-CoV-2 infection of variable disease severity and identified molecular signaling networks activated by glycocalyx components in immune and endothelial cells.

**Methods:**

We studied patients with RT-PCR confirmed COVID-19 pneumonia, patients with COVID-19 Acute Respiratory Distress Syndrome (ARDS) and healthy controls (wildtype, n=20 in each group) and measured syndecan-1, heparan sulfate and hyaluronic acid. The in-silico construction of signaling networks was based on RNA sequencing (RNAseq) of mRNA transcripts derived from blood cells and of miRNAs isolated from extracellular vesicles from the identical cohort. Differentially regulated RNAs between groups were identified by gene expression analysis. Both RNAseq data sets were used for network construction of circulating glycosaminoglycans focusing on immune and endothelial cells.

**Results:**

Plasma concentrations of glycocalyx components were highest in COVID-19 ARDS. Hyaluronic acid plasma levels in patients admitted with COVID-19 pneumonia who later developed ARDS during hospital treatment (n=8) were significantly higher at hospital admission than in patients with an early recovery. RNAseq identified hyaluronic acid as an upregulator of TLR4 in pneumonia and ARDS. In COVID-19 ARDS, syndecan-1 increased IL-6, which was significantly higher than in pneumonia. In ARDS, hyaluronic acid activated NRP1, a co-receptor of activated VEGFA, which is associated with pulmonary vascular hyperpermeability and interacted with VCAN (upregulated), a proteoglycan important for chemokine communication.

**Conclusions:**

Circulating glycocalyx components in COVID-19 have distinct biologic feedback effects on immune and endothelial cells and result in upregulation of key regulatory transcripts leading to further immune activation and more severe systemic inflammation. These consequences are most pronounced during the early hospital phase of COVID-19 before pulmonary failure develops. Elevated levels of circulating glycocalyx components may early identify patients at risk for microvascular injury and ARDS. The timely inhibition of glycocalyx degradation could provide a novel therapeutic approach to prevent the development of ARDS in COVID-19.

## 1 Introduction

Severe forms of COVID-19 associated with acute pulmonary failure (acute respiratory distress syndrome, COVID-19 ARDS) are a multisystemic, thrombotic and inflammatory disorder with high mortality (1, 2). Histopathological studies in COVID-19 ARDS showed wide spread endotheliitis involving multiple organs (3, 4) beside the affected lungs (5) which is in marked contrast to “classic” ARDS (6). This pathophysiologic difference is probably due to a combination of direct viral infection of endothelial cells (7) and the systemic inflammatory response of the organism (cytokine storm) (8) both resulting in vascular pathology (9, 10).

Under physiological conditions, vascular integrity is preserved by a network of glycocalyx proteins located at the interface between the endothelial cell surface and the vascular environment. Major components of the glycocalyx are hyaluronic acid and heparan sulfate. The endothelial transmembrane protein syndecan serves as connecting structure for both. Severe infection and inflammation lead to degradation of this protective layer by hyaluronidase and heparanase resulting in immunologically active hyaluronic acid and heparan sulfate fragments, both markers of glycocalyx shedding. In patients with COVID-19, elevated levels of these glycocalyx fragments were strongly associated with organ failures and increased inflammatory cytokine levels (11).

There is little information, however, regarding the mediators of intercellular communication between immune and endothelial cells and the molecular signaling cascades of glycocalyx fragmentation in COVID-19 pulmonary failure and microvascular injury.

An important part of intercellular communication are extracellular vesicles (EVs) (12). They play a major part in the regulation of immune response (13) in multiple human disorders such as pneumonia (14) or vascular pathology in general (15–17). In COVID-19, EVs are also key regulators of the immune response and disease progression (18–20).

EVs are categorized as either exosomes or ectosomes. Exosomes are of endosomal origin with a size between 40 to 160 nm whereas ectosomes are larger EVs generated by the direct outward budding of the plasma membrane with a diameter in the range between 50 nm to one µm in diameter.

EVs contain many components of their cells of origin including RNA, as well as DNA, lipids and cytosolic and cell-surface proteins (21). Mechanistic studies show that these molecules serve as biological signals transported by EVs from their cells of origin to specific target cells, underlining their important function in intercellular communication (12). Non-coding RNA - in particular - microRNAs (miRNAs) - as EV components are taken up by target cells where they regulate gene transcription.

In this study, we measured the circulating glycocalyx elements hyaluronic acid and heparan sulfate and its connecting transmembrane protein syndecan-1 along with the von Willebrand factor-cleaving protease ADAMTS13 in hospitalized patients with COVID-19 disease and identified molecular signaling networks targeted by these glycocalyx components and ADAMTS13. Network analysis was based on data from high-throughput RNA sequencing (RNAseq) of mRNA isolated from blood cells and miRNAs derived from EVs as mediators of intercellular communication between blood, endothelial cells and immune cells. Our aim was to characterize the role of circulating glycocalyx components in immune and endothelial cell activation in COVID-19.

## 2 Methods

**Figure 1:**
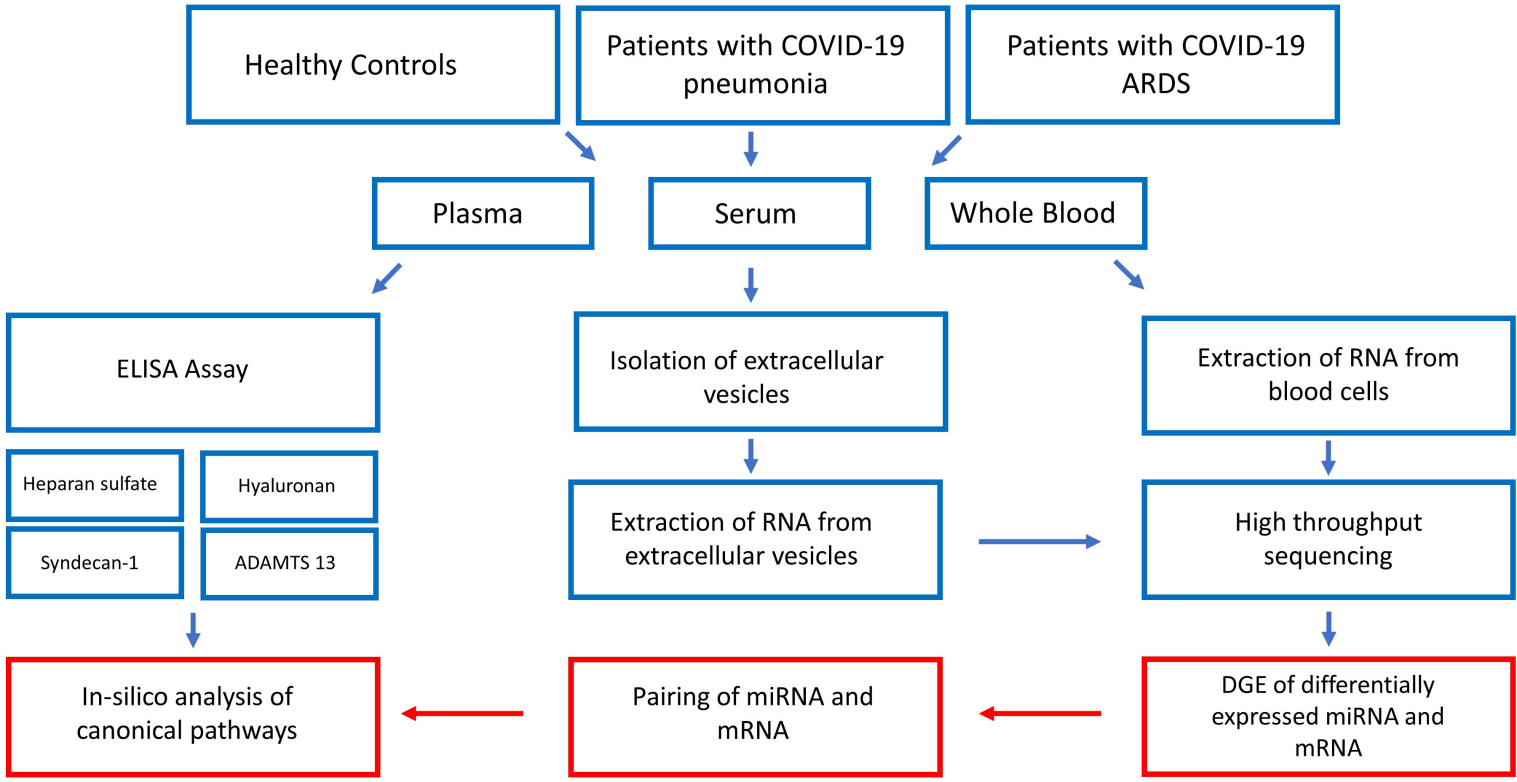
Step by step illustration of the study procedure. After recruitment of the study participants and blood sampling, plasma concentrations of glycocalyx components were measured by ELISA, EVs were extracted from serum and RNA extracted from EVs and whole blood cells followed by high-throughput RNA sequencing. The last step consisted of bioinformatic analysis resulting in the construction of glycocalyx component signaling networks in COVID-19. ADAMTS13 = von Willebrand factor-cleaving protease, DGE, differential gene expression analysis.

**Figure 1** gives a summarized overview of the methodological approach used in this study.

### 2.1 Study groups

A total of 60 individuals were studied at the Ludwig-Maximilians-University Hospital. The study cohort consisted of 20 healthy controls without known or suspected infection with SARS-CoV-2, 20 patients with COVID-19 pneumonia, and 20 patients with COVID-19 ARDS. COVID-19 patients had at least one positive nasal swap for SARS-CoV-2 virus (SARS-CoV-2-RNA PCR test, RdRP-Gen IP4) and typical symptoms. All patients were infected with the wildtype of SARS-CoV-2 during the first COVID-19 wave. Patients with COVID-19 pneumonia were recruited consecutively from the hospital COVID-19 isolation facility and ARDS patients from the COVID-19 ICUs of the Ludwig-Maximilians-University Hospital. Healthy controls were enrolled by advertisement and from hospital staff. All study participants were recruited between 03/2020 and 04/2020 and were non-vaccinated. Exclusion criteria for the study were: No consent given by patients or legal representative, age < 18, pregnancy, preexisting chronic infectious disorders (e.g., endocarditis, HIV or hepatitis), current tumor or malignant disorders, limited patient’s life expectancy < 6 months (independent of COVID-19 disease) and immunosuppression. Inclusion criteria for COVID-19 associated pneumonia were clinical symptoms like fever, cough or dyspnoea and a CURB-65 score ≥ 1. SARS-CoV-2 associated ARDS was diagnosed by bilateral chest radiographical opacities with severe hypoxemia due to non-cardiogenic pulmonary edema according to the Berlin ARDS definition (22). The final diagnosis of ARDS was made by experienced ICU and emergency room clinicians without prior knowledge of the results of the molecular studies.

### 2.2 Blood sampling

Blood samples from the COVID-19 pneumonia patients and healthy volunteers were obtained by venipuncture and from patients with COVID-19 ARDS by sampling from arterial lines. A previous study from our group has shown that venous vs. arterial blood sampling has very little effect on expression values of EVs derived miRNAs (23). Blood samples were obtained within the first 24 h after admission to the emergency room (COVID-19 pneumonia) or the ICU (COVID-19 ARDS). Five ml EDTA tubes and 9 ml Serum tubes were used for collection. Serum and EDTA samples were centrifuged at 3400 g for 10 minutes at 4°C and the supernatant stored at -80°C. Whole blood samples for cellular RNA extraction were collected in PAXgene tubes (PAXgene, Qiagen, Hilden, Germany) in accordance with the supplier’s protocol.

### 2.3 Isolation and characterization of extracellular vesicles

EVs from study participants were available from a previous study (18) and were precipitated by an isolation kit (miRCURY Exosome Isolation Kit, Qiagen, Venlo, Netherlands), characterized by Nanoparticle Tracking Analysis (ZetaView PMX 110, Particle Metrix, Meerbusch, Germany) and visualized by transmission electron microscopy as described earlier (23).

### 2.4 Measurement of glycocalyx components

Glycocalyx components (syndecan-1, hyaluronic acid and heparan sulfate) and ADAMTS13 were measured from plasma by ELISA, see supplemental information for details.

### 2.5 RNA processing and sequencing

Extraction and processing of non-coding RNA (miRNA) from EVs was performed as previously described (18). Long RNA (mRNA) sequencing from whole blood of all studied individuals was successful in 15 healthy volunteers, 19 patients with COVID-19 pneumonia and in 15 patients with COVID-19 ARDS. A blood RNA Kit (PAXgene RNA Kit, QIAGEN, Hildesheim, Germany) was used for the extraction of total RNA. All samples were processed according to the manufacturer´s protocol. The quality of the extracted RNA molecules was checked by RNA 6000 Nano assay on a Bioanalyzer 2100 (Agilent Technologies, Waldbronn, Germany). The extracted RNA was quantified using a ND-1000 NanoDrop (Thermo Fisher Scientific, Darmstadt, Germany) spectrometer.

For long RNAseq, one µg of total RNA was rRNA and globin depleted using the QIAseq FastSelect-rRNA/Globin Kit (Qiagen, Hildesheim, Germany) and the settings for RIN 5-6 and insert size 150-250 bases. Libraries were prepared with the QIAseq Stranded Total RNA library prep kit (Qiagen, Hildesheim, Germany) according to the manufacturer’s protocol. An adapter dilution of 1:20 was applied. Obtained DNA libraries were quantified and quality checked with the High Sensitivity DNA assay on a Bioanalyzer 2100 (Agilent Technologies, Waldbronn, Germany). Sequencing was performed on a HiSeq 2500 (Illumina Inc., San Diego, CA, USA) in two runs.

Small RNA sequencing, libraries were prepared with a NEBNext Multiplex Small RNA Library Prep Set for Illumina (New England Biolabs Inc., Ipswich, USA) as described previously (24). RNA data were processed by FASTQC software (https://www.bioinformatics.babraham.ac.uk/projects/fastqc/) and sequence quality and length distribution reviewed. In this way, adaptor sequences were trimmed and reads without adaptors were removed. Reads mapped to ribosomal and transfer RNA or sequences shorter than 16 nucleotides were deleted. Then sequences of the processed reads were matched with RNAcentral (https://doi.org/10.1093/nar/gkw1008). Finally, different gene expression between patient groups and healthy controls were identified by using the Bioconductor package DGEeq2 for R (R Foundation for Statistical Computing, Vienna, Austria, version 4.0.1). Thresholds were set at a log2fold change ≥|1|, an adjusted p-value (p_adj_) of ≤ 0.1 and a mean expression of ≥ 50 for significantly regulated protein-coding transcripts.

### 2.6 Statistical analysis of demographics and clinical data

The estimation of the number of patients (n_COVID-19_ = 40) required demonstrating statistically significant differences in plasma concentrations of glycocalyx components and heparanase activity was based on a previously published study, where the inclusion of 10 healthy controls and 48 COVID-19 patients revealed significantly higher heparanase and heparan sulfate concentrations in patients as compared to controls (25). Case number estimation for mRNA sequencing resulted from our earlier study in COVID-19 patients where the analysis of differential gene expression analysis data showed significant differences in EV miRNA expression levels when 20 healthy controls and 40 patients with either COVID-19 pneumonia or COVID-19 ARDS were studied (18). We assumed that an equal number of healthy controls and patients would again result in sufficient statistical power to reject the null hypothesis at an α-level of p = 0.05 that there is no significant difference in plasma concentrations of glycocalyx components and mRNA expression levels between controls and the two groups of patients.

Demographic, clinical data and differences in glycocalyx components and ADAMTS13 levels between groups were compared using the non-parametric Mann-Whitney U test and Bonferroni corrections were considered because of multiple comparisons between groups. The Chi-square or Fisher’s exact test was used for comparison of categorical variables. Data analysis was performed using Python (version 3.7, Python Software Foundation, Beaverton, USA) and R version R-3.6.2 (26). Data in the text and in tables are reported as median and interquartile range (IQR). All statistical tests were two-tailed and a p-value < 0.05 was considered statistically significant.

### 2.7 Pathway analyses

The resulting RNAseq data were analyzed using Ingenuity Pathway Analysis (IPA^®^, QIAGEN Digital Insights, Redwood, CA, USA) for *in-silico* identification of mRNA gene targets of miRNAs and the construction of causal networks involving immunological effects of the glycocalyx components hyaluronic acid, heparan sulfate, syndecan-1, the anti-thrombotic protein ADAMTS13, as measured by ELISA, and HYAL1 (hyaluronidase 1, from RNAseq). Significantly regulated miRNAs and mRNAs fulfilling predefined cut-off values (baseMean ≥50, log2FC ≥1 or log2FC ≤ −1 and p_adj_ ≤ 0.05 for miRNAs and p_adj_ ≤ 0.1 for mRNAs) were entered into the *IPA^®^ microRNA Target Filter* and conventionally paired for downregulated miRNAs in combination with their upregulated mRNAs and vice versa. As there is evidence that vascular pathology resulting from endothelial injury plays a major role in COVID-19 (8) along with the immunological reaction to the virus, IPA^®^ network generation was filtered to effects on immune and endothelial cells. Additional filter criteria were *respiratory disease* and *experimentally confirmed* or *highly predicted relationships* (see the online supplement for a detailed description of filter criteria). Network construction focused on comparisons between COVID-19 pneumonia and COVID-19 ARDS to healthy controls and COVID-19 pneumonia to COVID-19 ARDS. These comparisons were performed to identify the regulatory role of the glycocalyx components and their degradation products in different disease severities and to illustrate the progression from pneumonia to COVID-19 ARDS.

### 2.8 Ethics approval and consent to participate

Approval of the study was granted by the Ethics Committee of the Medical Faculty of the Ludwig‐Maximilians‐University of Munich under protocol #18-398. All samples were pseudonymized during analyses. The study was conducted in accordance with the declaration of Helsinki and written informed consent to participate was obtained from each participant or the patient’s legal representative.

## 3 Results

### 3.1 Study population

Twenty individuals were studied in each of the three study groups. Their demographic and clinical data are presented in **Table 1**. Healthy controls were significantly younger and showed a significantly lower body mass index (BMI) than patients. Age and BMI did not differ significantly between patients with COVID-19 pneumonia or COVID-19 ARDS. Patients with ARDS showed significantly higher levels of inflammatory parameters (IL-6, C-reactive protein, leucocyte count, procalcitonin) at admission to the ICU and required a significantly longer hospital stay. Eight patients with COVID-19 pneumonia at hospital admission developed severe pulmonary failure (ARDS) during treatment at the COVID-19 isolation facility and required intubation, mechanical ventilation and transfer to the ICU. One patient died in the COVID-19 pneumonia group, another patient who was originally admitted with COVID-19 pneumonia developed ARDS and died during ICU treatment and a further patient deceased after direct admission to the ICU from COVID-19 ARDS.

**Table 1:** Comparison of demographic and clinical data between study groups.

Group	Healthy Controls	COVID-19 Pneumonia	COVID-19 ARDS
**Age**	35 (31 - 39)^§,&^	63.5 (53.5 - 75.5)	64.5 (55 - 71)
**BMI (kg/m^2^)**	23.4 (21.8 - 25.6) ^§,&^	26.6 (24.5 - 33.3)	28.2 (25.7 - 31.8)
**Sex, n (f/m)**	8/12	2/18	2/18
**Hospital Stay (d)**		16.5 (20.0-40.0) ^&^	31 (20.25 - 47.5)
**PCT (ng/ml)**		0 (0 - 0.2) ^&^	0.9 (0.375 - 1.05)
**Leukocyte count (G/l)**		5.045 (3.24 – 8.0) ^&^	8.675 (7.555 - 12.03)
**CRP (mg/dl)**		5 (2 - 10.53) ^&^	22.5 (14.5 - 28.6)
**IL-6 (pg/ml)**		43.2 (16.9 - 82.42) ^&^	304.5 (112.2 - 575.3)
**ICU stay (d)**			21 (14 - 37)
**Progression to ARDS (n)**		8	
**CURB Pneumonia Score**		1 (0 - 2)	
**SOFA - Score**			10 (9 - 11)
**P_a_O_2_/F_i_O_2_**			136 (97.85 - 158.25)

CURB Score = a score based on confusion, urea, respiratory rate, blood pressure, and age to quantify the severity of community acquired pneumonia (28). P_a_O_2_/F_i_O_2_ = ratio of partial pressure of oxygen in blood (P_a_O_2_) and the fraction of oxygen in the inhaled air (F_i_O_2_). Used as a rough measure of oxygenation in patients on ventilators, particularly in ARDS. SOFA Score = Sequential Organ Failure Assessment score. Well validated and widely used score for quantification of disease severity in ICU patients with sepsis (29). ^§^significantly different compared to pneumonia (p<0.05); &significantly different compared to ARDS (p<0.05). Data are median and quartiles. IL-6 = interleukin-6, CRP = C-reactive protein, BMI = body mass index, PCT = procalcitonin, a widely used and validated biomarker in ICU patients (27).

### 3.2 Plasma concentrations of glycocalyx components and the von Willebrand factor-cleaving protease ADAMTS13

**Figure 2:**
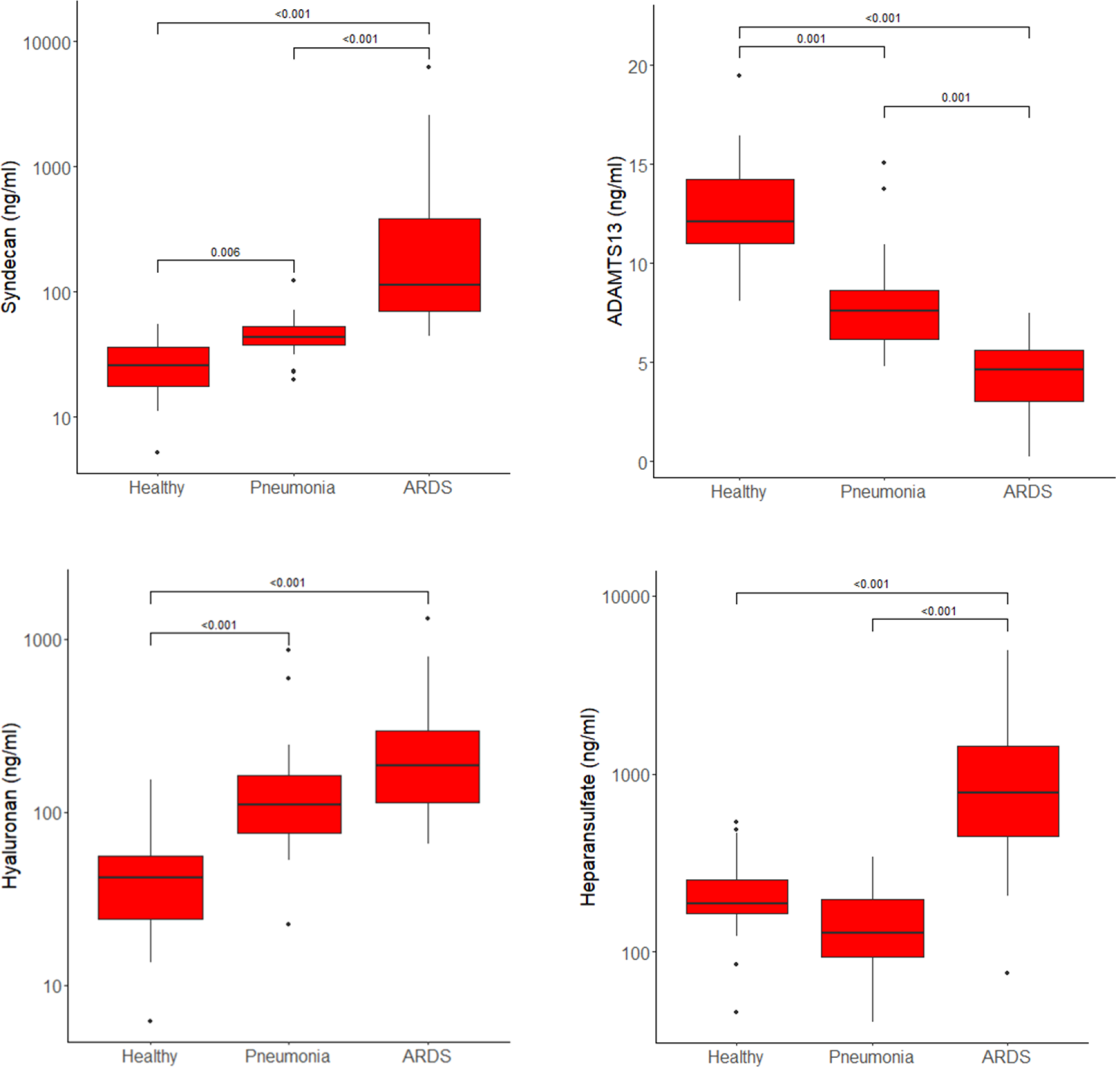
Plasma concentrations of syndecan-1, ADAMTS13, hyaluronic acid and heparan sulfate in healthy controls, patients with COVID-19 pneumonia and those who progressed to ARDS presented as boxplots. Horizontal lines across the boxes show significant differences with p-values between groups. Boxes represent Q1 and Q3 with median in between. Whiskers are Q1-1.5*IQR and Q3+1.5*IQR. Outliers are shown as dots.

The highest plasma concentrations of syndecan-1, heparan sulfate and hyaluronic acid and the lowest levels of ADAMTS-13 were measured in patients with COVID-19 ARDS. Heparan sulfate plasma concentrations did not differ between healthy controls and patients with COVID-19 pneumonia, but ARDS patients showed significant higher levels of heparan sulfate compared to the other study groups (**Figure 2**).

When analyzing the difference between pneumonia patients who later progressed to ARDS (n=8 in the pneumonia group) and those who did not show a further progression of the disease, hyaluronic acid plasma levels were significantly higher in the subgroup with disease progression (164, 126 - 211 ng/ml vs. 80, 64 - 110 ng/ml, p=0.005, median and IQR).

The absolute values for each study group for all measured glycocalyx components and ADAMTS13 are shown in **Table E 1** in the **Supplementary File**.

### 3.3 RNA processing and sequencing

The comparison of RNAseq data by DGE analysis between healthy volunteers (baseline) and patients with COVID-19 pneumonia revealed 42 differentially regulated miRNAs isolated from EVs (17 upregulated) (**s. Table E 2** in the **Supplemental Information** for miRNAs) and 3448 significantly regulated mRNA transcripts (1556 upregulated). The corresponding analysis of healthy volunteers vs. patients with COVID-19 ARDS uncovered 72 significantly regulated miRNAs (30 upregulated) (**s. Table E 3** in the **Supplement**) and 2374 significantly regulated mRNAs (854 upregulated). The analogous assessment of COVID-19 pneumonia in comparison to COVID-19 ARDS showed 20 significantly regulated miRNAs (5 upregulated) (**s. Table E 4** in the **Supplement**) and 149 mRNA transcripts with significantly different expression values (53 upregulated). mRNA sequencing data for all groups are presented in the online data deposition of the study (European Nucleotide Archive, access number to be determined).

### 3.4 Molecular networks of glycocalyx components and ADAMTS13 signaling

#### 3.4.1 Signaling network of COVID-19 pneumonia compared to the healthy state

**Figure 3:**
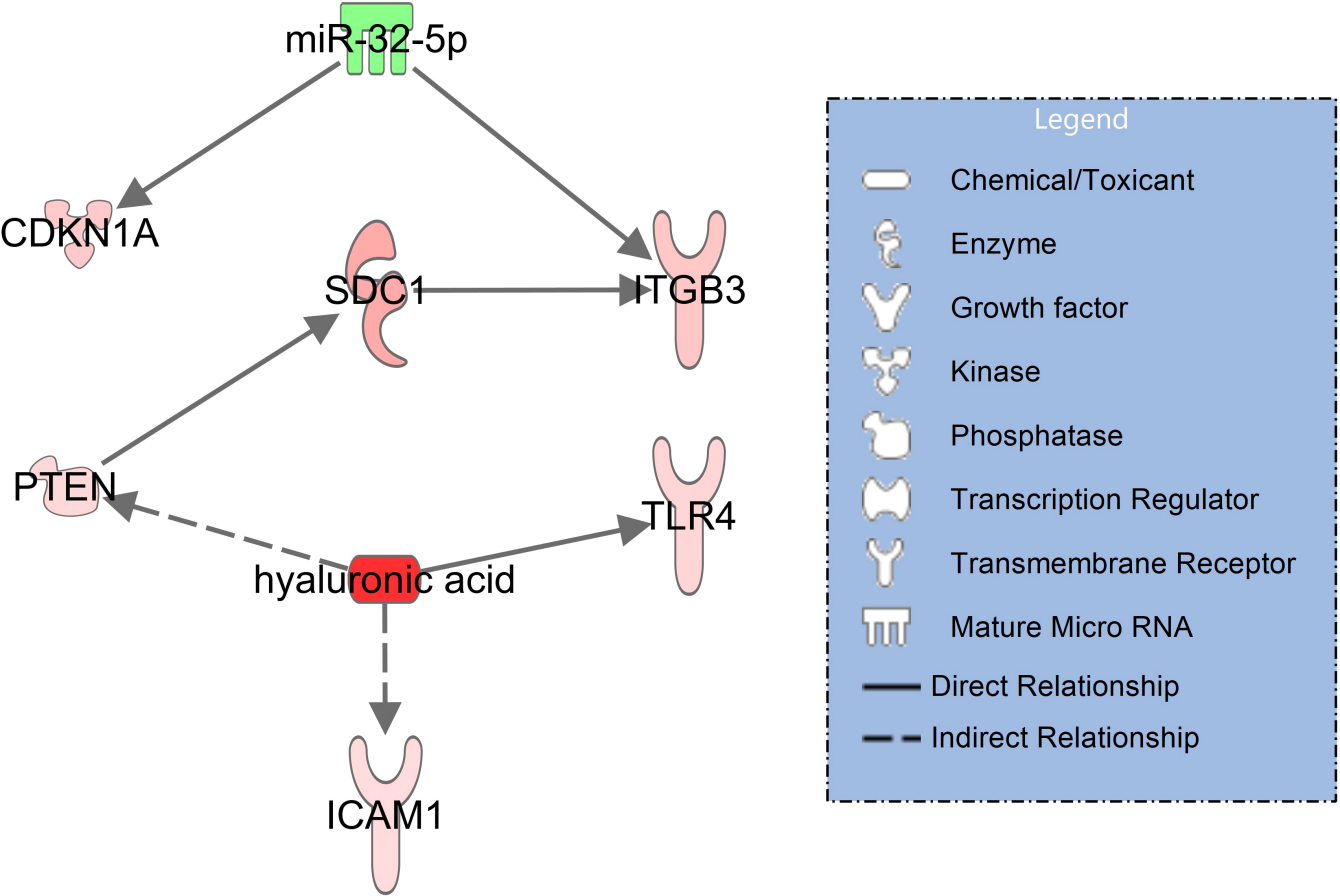
Network illustrating hyaluronic acid and syndecan-1 (SDC1) signaling in immune and endothelial cells for patients with COVID-19 pneumonia with healthy controls serving as baseline. RNAseq data resulted from miRNAs (extracellular vesicles) and mRNA transcripts (blood cells). The significantly downregulated miR-32-5p is shown in green and the activated molecules SDC1 and hyaluronic acid are shown in red. The network was filtered for experimentally observed findings only, solid lines indicate direct and dashed lines indirect relationships.

In the signaling network of syndecan-1 and hyaluronic acid (**Figure 3**), upregulated syndecan-1 (SDC1, log2FC=1.76, padj = 0.034) interacts with upregulated ITGB3 (log2FC=1.19, padj=0.005) (30) which is also activated by downregulated miR-32-5p (log2FC=-1.25, padj=0.007). Hyaluronic acid activates TLR4 (log2FC=0.91, padj<0.001) along with ICAM-1 (log2FC=0.77, padj=0.014) (31) and indirectly PTEN (log2FC=0.85, padj=0.002) (32) which in turn interacts with SDC1 (33).

### 3.4.2 Signaling network of COVID-19 ARDS compared to the healthy state

**Figure 4:**
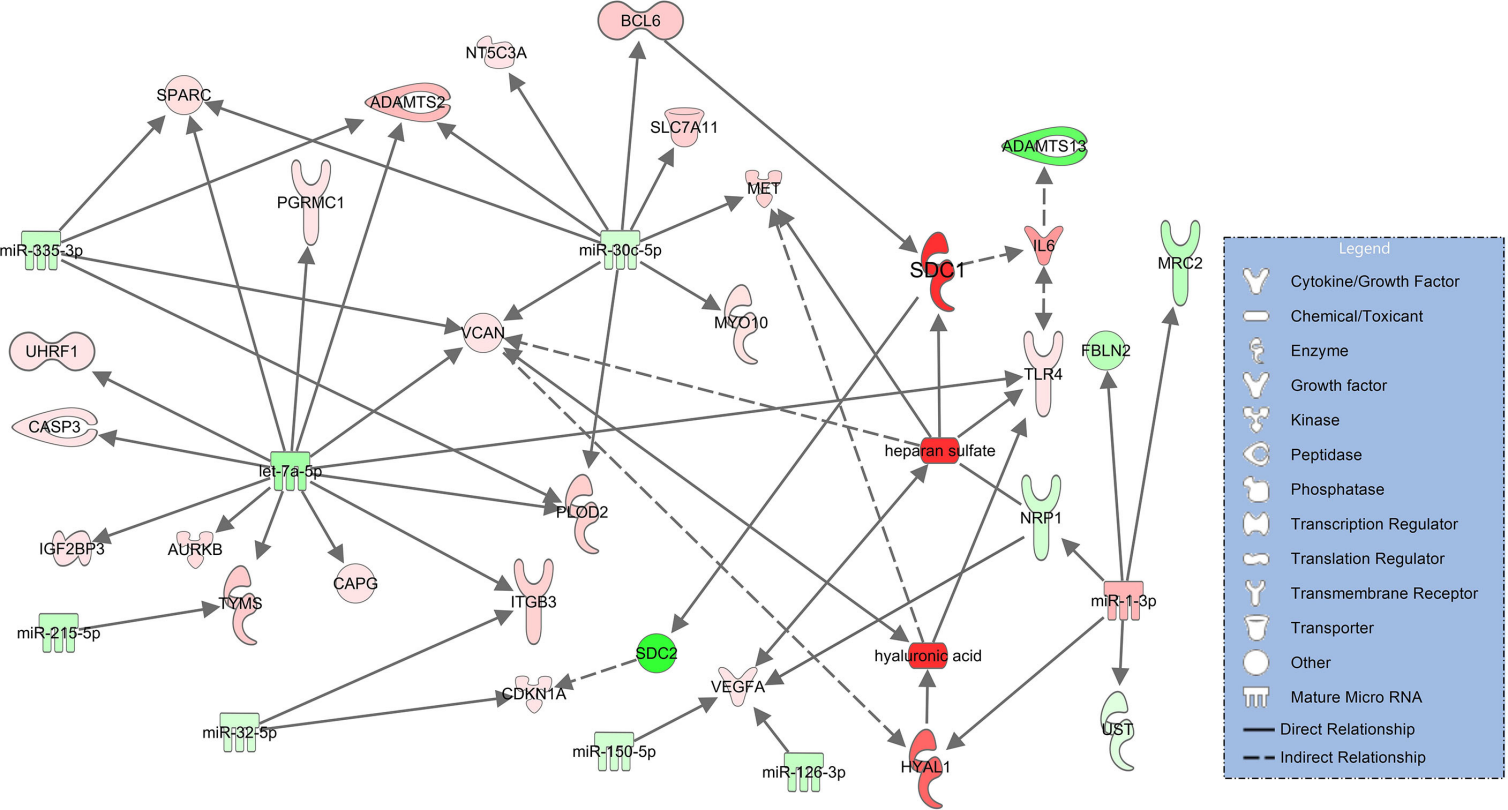
Network for immune and endothelial cells comparing COVID-19 ARDS to the healthy state based on experimentally observed findings. Upregulated molecules are shown in red and downregulated ones in green. Significantly increased plasma concentrations of hyaluronic acid, heparan sulfate, syndecan-1 (SDC1) and downregulated signaling of ADAMTS13 are colored in darker red/green. Solid lines indicate direct and dashed lines indirect relationships.

In severe pulmonary failure (ARDS), syndecan-1 (SDC1) upregulates IL-6 (34) and downregulates SDC2 (syndecan-2, log2FC=-13.2, p_adj_<0.001) (35). Elevated IL-6 levels in COVID-19 ARDS (**s. Table 1**) downregulate ADAMTS13 (36). Heparan sulfate binds to downregulated NRP1 (neurophilin-1, log2FC=-3.14, p_adj_=0.091) (37) and upregulated VCAN (versican, log2FC=2.43, p_adj_<0.001) (38) which also interacts with hyaluronic acid (39). HYAL1 (hyaluronidase 1), the degradation enzyme of hyaluronic acid, is upregulated in the network and targets VCAN (38) along with hyaluronic acid. Degradation products of hyaluronic acid (40) and heparan sulfate (41) are associated with upregulation of MET (tyrosine-protein kinase Met, log2FC=3.92, p_adj_=0.062) (**Figure 4**).

#### 3.4.3 Signaling network of COVID-19 pneumonia after progression to COVID-19 ARDS

**Figure 5:**
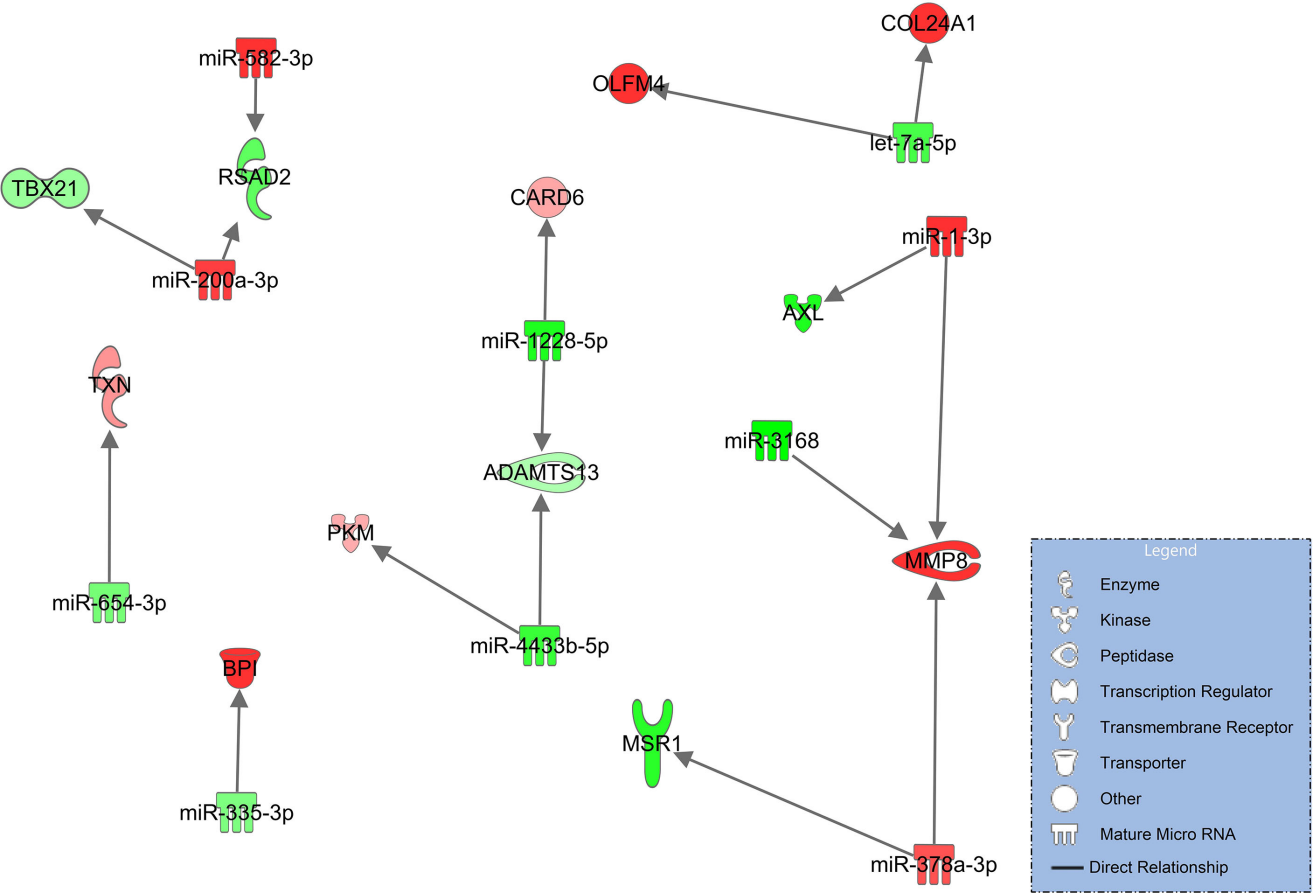
Immune and endothelial cell signaling in COVID-19 pneumonia compared to patients progressing to COVID-ARDS. Upregulated molecules are shown in red and downregulated in green. Network is based on experimentally observed findings and highly predicted interactions.

In this comparison, syndecan-1 and heparan sulfate plasma concentrations are significantly higher and ADAMTS13 significantly lower in patients progressing from pneumonia to severe pulmonary failure (ARDS) (**Figure 2**). The corresponding network derived from immune and endothelial cell signaling is considerably smaller than the networks comparing COVID-19 patients to healthy controls (**Figures 3**, **4**) and no direct regulatory effects for the glycocalyx components or ADAMTS13 can be identified (**Figure 5**). The downregulated miRNAs miR-1228-5p (log2FC=-2.29, p_adj_<0.001) and miR-4433-3p (log2FC=-2.63E-06) target ADAMTS13 and are predicted by the model to activate the interferon inducible CARD6 (log2FC=0.63, p_adj_=0.07, caspase recruitment domain family member 6) as well as upregulated PKM (log2FC=0.59, p_adj_=0.011, pyruvate kinase isozyme) but do not directly interact with ADAMTS134.

## 4 Discussion

Severely affected COVID-19 patients are in a state of endothelial injury with widespread microthrombosis coupled with hyperactivation of the immune system (10). Immune cell activation is reflected by high levels of IL-6, IL-1β, IL-18 known as the COVID-19 *cytokine storm* (42). High concentrations of pro-inflammatory cytokines in-turn lead to endothelial cell activation. The reduced levels of ADAMTS13 as seen in our study result in high concentrations of the endothelial adhesion protein von Willebrand Factor on the “naked” endothelial cell surface along with loss of its protective glycocalyx layer. This leads to platelet wall adhesion with microangiopathy and microthrombosis (43).

Less investigated, however, was the direct effect of circulating glycocalyx components and the decrease in ADAMTS13 on molecular signaling in immune and endothelial cells. We addressed this research question in our study and showed that circulating glycocalyx components during SARS-CoV-2 infection are not biologically inactive markers of severe microvascular injury but have distinct feedback effects on signaling networks in immune and endothelial cells resulting in immune and endothelial cell activation with an increase in inflammation and further enhancement of microthrombosis formation.

The construction of glycocalyx component signaling networks in our study was based on RNA expression levels determined by high-throughput sequencing of miRNAs from EVs and mRNA transcripts from blood cells. Extracellular vesicles as a source for miRNAs were selected because our group and others have demonstrated their important role in regulating the immune response and the development of immunothrombosis (18, 20). In addition, platelets release extracellular vesicles with procoagulant activity in COVID-19 (44). miRNAs associated with extracellular vesicles can regulate mRNAs transcription in target cells during systemic inflammation and in vascular disorders such as atherosclerosis (17, 45, 46).

When comparing plasma concentrations of syndecan-1 and hyaluronic acid in COVID-19 pneumonia to healthy controls, both glycocalyx components were significantly elevated (**Figure 2**). The corresponding molecular signaling networks demonstrated that several important mRNA transcripts are regulated by these glycocalyx components in immune and endothelial cells. Syndecan-1 interacts directly with ITGB3 (CD61 or integrin beta 3) (**Figure 3**). Additionally, ITGB3 is activated by downregulated miR-32-5p. In COVID-19, ITGB3 is indicative of a large number of megakaryocytes in the pulmonary circulation (47). Megakaryocytes as the source of platelets (48) lead to an increase risk for platelet activated thrombus formation. Furthermore, ACE2 expression (angiotensin−converting enzyme 2) as the key host protein of COVID-19, correlates with expression levels of ITGB3 in post-mortem lung tissues obtained from patients who died from COVID-19 ARDS (49). In our study, expression in extracellular vesicles is downregulated in COVID-19 patients in comparison to healthy controls. Downregulation of miR-32-5p showed potential activating effects on ACE2 expression in a recent in-silico investigation (50). In combination with signaling effects of syndecan-1, a lower expression of miR-32-5p could therefore be associated with immune cell activation, ACE2 mediated virus entry and pulmonary microthrombi formation.

A further important finding in the COVID-19 pneumonia network was the direct interaction between hyaluronic acid with TLR4 (toll-like receptor 4) (51) (**Figure 3**). TLR4 activation results in increased ACE2 expression on the cellular surface of circulating monocytes leading to increased susceptibility to infection with SARS-CoV-2. Extracellular vesicles (exosomes) were found as a source of ACE2 in this context (52). Blocking of TLR4 signaling has been suggested as a means of limiting pulmonary injury in viral infections (53).

The significant increase in syndecan-1 and hyaluronic acid, the decrease in ADAMTS13 and the associated activation of procoagulant and inflammatory pathways seen in COVID-19 pneumonia indicates that patients with this clinically milder variety of COVID-19 disease are also at risk of microvascular complications.

As a next step, we constructed glycocalyx component and ADAMTS13 signaling networks which compared patients with COVID-19 ARDS - the most severe form of the disease - to healthy controls. In this analysis, all glycocalyx components are significantly higher and ADAMTS13 levels are even lower than in COVID-19 pneumonia, a reflection of the progress in disease severity towards pulmonary failure. In the resulting molecular network, the activating effect of heparan sulfate on TLR4 is maintained and, in addition, this proteoglycan is associated with an increase in VEGFA signaling as heparan sulfate is an endogenous agonist on VEGFA and TLR4 (54–56). VEGFA acts as a potent inductor of vascular leakage (55), a hallmark of pulmonary injury leading to ARDS. The signaling network involving TLR4 and VEGFA in COVID-19 ARDS is more complex than in pneumonia and shows additional activating effects of downregulated miR-150-5p and miR-126-3p on VEGFA. Downregulation of miR-150-5p is associated with severe COVID-19 disease (57). miR-126-3p amongst other miRNAs differentiated between survivors and non-survivors in patients with COVID-19 ARDS in an earlier study (58). Another interesting connection in this network is the binding of heparan sulfate to downregulated NRP1 (neurotropin 1 or neuropilin-1), an antagonist of VEGF (59). Lower expression of NRP1 could result in even higher activity of VEGF, further enhanced by additional downregulation of NRP1 through upregulated miR-1-3p derived from extracellular vesicles in our data (**Figure 4**). Upregulated miR-1-3p in bronchial aspirates also distinguished between survivors and non-survivors with COVID-19 ARDS (58).

This analysis was followed by comparing patients with pneumonia to those who had developed severe ARDS. Syndecan-1 and heparan sulfate plasma concentrations were significantly higher and ADAMT13 levels significantly lower than seen in COVID-19 pneumonia (**Figure 2**) but no direct regulatory effects of these molecules in the network could be identified. In addition, this network (**Figure 5**) was smaller and more fragmented than the signaling cascades comparing the healthy state to pneumonia and ARDS. This indicates that many pathophysiologic changes occur during the early phase of the disease when COVID-19 has not yet progressed to pulmonary failure. In the network, ADAMTS13 was targeted by downregulated miR-1228-5p and miR-4433b-5p (**Figure 5**). As ADAMTS13 levels were already low, counter regulatory effects of these miRNAs could explain this finding; the inhibition of ADAMTS13 most likely occurs by other mechanisms not reflected by the network. It is of interest to note, however, that miR-1228-5p was upregulated in patients with COVID-19 pneumonia (**s. Table E 2** in the online supplement) in contrast to the comparison between COVID-19 pneumonia to ARDS where this EV-derived miRNA was downregulated (**Figure 5**). This points to the possibility that miR-1228-5p might be involved in an activation of ADAMTS-13 in COVID-19 ARDS. miR-1228-5p also upregulated CARD6 (caspase recruitment domain family, member 6) which plays a role in immune defense, but the exact pathophysiologic function of CARD6 is poorly defined (60). Nevertheless, CARD6 is highly expressed in neutrophils, endothelial progenitor and venous endothelial cells (**s. Figure E 2** in the **supplement**). miR-4433b-5p upregulated PKM (pyruvate kinase isozyme). Pyruvate kinase is responsible for net ATP production within the glycolytic sequence. In contrast to mitochondrial respiration, energy regeneration by pyruvate kinase is independent from oxygen and allows survival of organs under hypoxic conditions, which are present in ARDS. The highest expression of PKM in COVID-19 ARDS patients is found in circulating mono-CD14^+^ cells (61).

Interestingly, the subgroup of patients in our study originally admitted with pneumonia who later progressed to ARDS showed significantly higher levels of hyaluronic acid, while hyaluronic acid plasma levels of all pneumonia patients were not statistically significantly different to ARDS patients.

A limitation of our findings results from the fact that our study is missing a proteomics analysis of blood and additional cellular studies of blood and endothelial cells but our findings can form the basis for further proteomic and cell-based analyses of glycocalyx signaling networks and suggests a novel mechanism for immune modulation by glycocalyx components involving EV-derived miRNAs. Furthermore, the regulatory consequences of glycocalyx components and ADAMTS13 on immune and endothelial cells identified by the networks were not directly experimentally confirmed in our data set. The canonical pathways identified in our study were based on the *Knowledge Base* item underlying the IPA^®^ software. This *Knowledge Base* is created by millions of manually curated data obtained from scientific journals, publicly available molecular content databases, textbooks and more and allows the query, visualization and computation across the *Knowledge Base* in relationship to the researchers own dataset of miRNA, mRNA and protein findings uploaded into IPA^®^ (62). This holistic approach has been applied to COVID-19 in an earlier study (63) and results in networks which allow the identification of signaling cascades not previously studied. As a disadvantage, the regulatory effects of glycocalyx components in our networks could not explicitly be proven as causal and some of the interactions could still be correlational. However, most regulatory effects underlying the network connections were experimentally confirmed in earlier experimental studies or showed at least a high biologic plausibility.

## 5 Conclusion

Circulating glycocalyx components in COVID-19 are not harmless byproducts of microvascular injury but result in further activation of immune and endothelial cells and may fuel a positive feedback loop resulting in immune activation and more severe systemic inflammation leading to microcirculatory failure. These effects already appear during the early phase of COVID-19 when patients present with pneumonia prior to progression to pulmonary failure and ARDS. The timely identification of patients at risk for ARDS is therefore critical and glycocalyx component levels may serve as biomarkers for this negative outcome. A therapeutic consequence for COVID-19 patients with early evidence of microvascular injury would then be the targeted use of antiviral agents (e.g., Ritonavir-boosted nirmatrelvir or Remdesivir) or the timely administration of pharmacologic compounds that protect the glycocalyx layer (10).

## Ethics statement

The studies involving human participants were reviewed and approved by Ethics Committee of the Medical Faculty of the Ludwig‐Maximilians‐University of Munich. The patients/participants provided their written informed consent to participate in this study.

## Author contributions

All authors contributed to the study conception and design. Material preparation and data collection was performed by MB, FB, AM and MK. ELISA-was performed by MB. Molecular analysis was executed by MR and BK. Data analysis was done by AM, MB, FB, BK and J-NB. AM, MR, MP and GS worked on the manuscript. All authors commented on previous versions of the manuscript. All authors contributed to the article and approved the submitted version.
