# Supplementary material for: Extensive blood transcriptome analysis reveals cellular signaling networks activated by circulating glycocalyx components reflecting vascular injury in COVID-19
Source: Front Immunol. 2023 Jan 26;14:1129766. doi: 10.3389/fimmu.2023.1129766 (PMC9909741; doi:10.3389/fimmu.2023.1129766)
Supplement: Supplementary file 1 [file DataSheet_1.docx]

**Detailed description of glycocalyx measurements by ELISA**

**Syndecan-1**

Syndecan-1 protein was measured using the sandwich ELISA from Diaclone (Besançon cedex, France). Samples and standard solutions were prepared according to the manufacturer's instructions. On the microplate, which was already coated with antibodies, 100µl of the prepared samples or standard solutions were placed per well. This was followed by incubation with the anti-Syndecan 1 biotinylated antibody. After washing, the substrate was added. The reaction was stopped by adding a H2SO4 stop reagent. The evaluation was performed by an ELISA reader at 405 nm. An XY-curve standard curve was prepared for each series of experiments.

**ADAMTS 13**

The Quantikine human ADAMTS-13 ELISA (R&D Systems Inc., Minneapolis, USA) was used to measure ADAMTS13 (A Disintegrin and Metalloproteinase with a Thrombospondin Type 1 motif, member 13, Von Willebrand factor-cleaving protease) levels. 50 µl of sample material was used per well. After the incubation period was completed, a wash was performed. In the next step, 200µl of horseradish peroxidase conjugated polyclonal antibody was added. After the incubation period had expired, the washing procedure was repeated. After addition of substrate solution for 30 minutes, a color change occured. The reaction was stopped using a stop solution containing sulfuric acid. Finally, the optical density was measured within 30 minutes using an ELISA reader set to 405nm. For each set of samples examined, a standard curve was generated using computer software capable of generating a logistic curve fit with four parameters (s. Figure e 1).


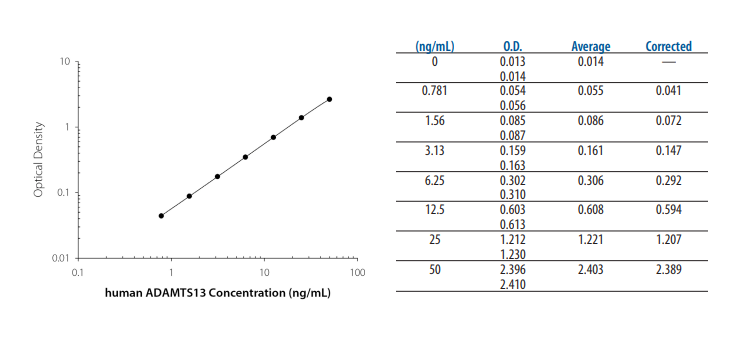


**Figure e 1**: Logistic curve fit for ADAMTS13 ELISA measurement.

**Hyaluronan (hyaluronic acid)**

Hyaluronan levels were determined by the Hyaluronan ELISA kit from Echelon (Product number K-1200, Salt Lake City, USA). The Hyaluronan ELISA kit was a competitive ELISA. The microplate was coated with an antibody against hyaluronan. After adding the sample, the hyaluronan is bound by this antibody which blocks the bound antibody for further reactions. In a second step, a conjugated antibody is added, which forms a bond with the fixed antibodies that have not bound hyaluronan. After removal of the fluid, a substrate solution is added, and the optic density is determined using an ELISA reader (wavelength at 405 nm). It should be noted that only the antibody complexes that have not bound hyaluronan cause a color change. Therefore, the measured optical density is inversely proportional to the concentration of hyaluronan in the sample.

**Heparan sulfate**

For the determination of heparan sulfate plasma levels the competitive HS ELISA kit (antikörper-online.de, Aachen, Germany) was used. This kit contains a microplate already coated with antibodies against human heparan sulfate. After appropriate preparation, the samples were transferred to the microplate together with a horseradish-peroxidase (HRP) conjugated heparin sulfate. A competitive reaction followed in which the heparan sulfate contained in the plasma samples reacted with the peroxidase-conjugated heparin sulfate around the binding sites of the heparan sulfate fixed on the microplate. The higher the concentration of heparan sulfate in the samples, the less the HRP conjugated antibody is bound. After incubation, the excess antibodies and sample material were removed and the substrate for the HRP conjugated antibody was added. The color reaction was then stopped with a stop solution and the color intensity was measured at a wavelength of 450 nm. The measured absorbance is inversely proportional to the concentration of heparan sulfate in the sample. A four-parameter logistic standard curve was prepared for each series of experiments.

**Table E 1**: Values of glycocalyx components and ADAMTS13 concentrations in the study groups. All values are ng/ml.

|  | **Healthy** | **Pneumonia** | **ARDS** |
| --- | --- | --- | --- |
| **Syndecan-1** | 26 (18 - 37 ) | 43 (38 – 52)^§^ | 113 (70 - 387)^§^ |
| **ADAMTS13** | 12 (11 - 14 ) | 7.63 (6 - 8) ^§^ | 5 (3 – 6) ^§^ |
| **Hyaluronic acid** | 42 (24 – 56) | 110 (76 - 162) ^%^ | 185 (114 - 294) ^%^ |
| **Heparan sulfate** | 187 (165 – 254) | 127 (94 – 197) | 779 (448 - 1441)^$^ |

^§^significant difference in concentrations in comparison to healthy controls and between pneumonia and ARDS. ^&^significantly lower concentrations in pneumonia vs. ARDS and in comparison to healthy controls.

^%^significantly higher concentrations in pneumonia and ARDS vs. healthy controls.

^$^significantly higher concentrations in COVID-19 ARDS compared to pneumonia and healthy controls.

**Filters applied to network construction in IPA analysis**

Immune Cells

(tissues = Activated CD56bright NK cells OR Activated CD56dim NK cells OR Activated Vd1 Gamma-delta T cells OR Activated Vd2 Gamma-delta T cells OR Activated helper T cells OR B lymphocytes not otherwise specified OR BDCA-1+ dendritic cells OR BDCA-3+ dendritic cells OR Bone marrow-derived dendritic cells OR Bone marrow-derived macrophages OR CD34+ cells OR CD4+ T-lymphocytes OR CD56bright NK cells OR CD56dim NK cells OR Central memory cytotoxic T cells OR Central memory helper T cells OR Cytotoxic T cells OR Dendritic cells not otherwise specified OR Effector T cells OR Effector memory RA+ cytotoxic T cells OR Effector memory cytotoxic T cells OR Effector memory helper T cells OR Eosinophils OR Granulocytes not otherwise specified OR Immature monocyte-derived dendritic cells OR Immune cells not otherwise specified OR Intraepithelial T lymphocytes OR Langerhans cells OR Lymphocytes not otherwise specified OR Macrophages not otherwise specified OR Mast cells OR Mature monocyte-derived dendritic cells OR Memory B cells OR Memory T lymphocytes not otherwise specified OR Microglia OR Monocyte-derived dendritic cells not otherwise specified OR Monocyte-derived macrophage OR Monocytes not otherwise specified OR Mononuclear leukocytes not otherwise specified OR Murine NKT cells OR Myeloid dendritic cells OR NK cells not otherwise specified OR Naive B cells OR Naive helper T cells OR Natural T-regulatory cells OR Neutrophils OR Other B lymphocytes OR Other Dendritic cells OR Other Granulocytes OR Other Immune cells OR Other Lymphocytes OR Other Macrophages OR Other Memory T lymphocytes OR Other Monocyte-derived dendritic cells OR Other Monocytes OR Other Mononuclear leukocytes OR Other NK cells OR Other Peripheral blood leukocytes OR Other T lymphocytes OR PBMCs OR Peripheral blood leukocytes not otherwise specified OR Peripheral blood lymphocytes OR Peripheral blood monocytes OR Peritoneal macrophages OR Plasma cells OR Plasmacytoid dendritic cells OR Pre-B lymphocytes OR Pro-B lymphocytes OR T lymphocytes not otherwise specified OR Th1 cells OR Th17 cells OR Th2 cells OR Thymocytes OR Vd1 Gamma-delta T cells OR Vd2 Gamma-delta T cells) AND

(diseases = Respiratory Disease) AND

(confidence = Experimentally Observed) AND

(Expression Pairing = microRNA up, mRNA down OR microRNA down, mRNA up)

Endothelial Cells

(tissues = Endothelial cells not otherwise specified OR HUVEC cells OR Microvascular endothelial cells OR Other Endothelial cells) AND

(diseases = Respiratory Disease) AND

(confidence = Experimentally Observed) AND

(Expression Pairing = microRNA up, mRNA down OR microRNA down, mRNA up)

**Table E 2**: Significantly regulated miRNAs coprecipitating with extracellular vesicles. Comparison of healthy volunteers (baseline) to patients with COVID-19 pneumonia (Meidert et al., 2021).

| **miRNA** | **Expr Log Ratio** | **Expr p-value** | **Expr False Discovery Rate (q-value)** |
| --- | --- | --- | --- |
| miR-1228-5p | 2.916 | 3.89E-06 | 9.87E-05 |
| miR-542-3p | 2.378 | 5.15E-12 | 3.80E-09 |
| miR-3168 | 2.278 | 2.89E-05 | 4.63E-04 |
| miR-338-5p | 1.877 | 4.30E-09 | 3.52E-07 |
| miR-197-3p | 1.707 | 4.22E-11 | 1.56E-08 |
| miR-335-5p | 1.695 | 6.35E-07 | 2.34E-05 |
| miR-193a-5p | 1.624 | 2.34E-10 | 4.31E-08 |
| miR-221-3p | 1.521 | 2.90E-08 | 2.14E-06 |
| miR-502-3p | 1.437 | 5.54E-08 | 3.71E-06 |
| miR-450b-5p | 1.240 | 4.41E-05 | 5.93E-04 |
| miR-223-3p | 1.237 | 1.88E-04 | 1.92E-03 |
| miR-146a-5p | 1.226 | 2.83E-06 | 8.01E-05 |
| miR-1-3p | 1.204 | 3.76E-03 | 2.11E-02 |
| miR-378a-3p | 1.167 | 2.85E-05 | 4.63E-04 |
| miR-20a-5p | 1.161 | 1.03E-05 | 2.22E-04 |
| miR-1246 | 1.136 | 1.55E-04 | 1.69E-03 |
| miR-501-3p | 1.095 | 1.33E-06 | 4.45E-05 |
| miR-144-5p | -1.041 | 9.44E-04 | 7.17E-03 |
| miR-99b-5p | -1.068 | 1.07E-05 | 2.26E-04 |
| miR-409-3p | -1.071 | 8.71E-03 | 4.04E-02 |
| miR-146b-5p | -1.093 | 2.44E-07 | 1.38E-05 |
| miR-139-5p | -1.136 | 2.63E-05 | 4.63E-04 |
| miR-363-3p | -1.162 | 6.45E-04 | 5.34E-03 |
| miR-32-5p | -1.252 | 8.69E-04 | 6.74E-03 |
| miR-543 | -1.281 | 1.64E-03 | 1.11E-02 |
| miR-370-3p | -1.364 | 9.29E-04 | 7.13E-03 |
| miR-493-3p | -1.373 | 3.57E-03 | 2.09E-02 |
| miR-654-3p | -1.384 | 1.75E-04 | 1.81E-03 |
| miR-374a-5p | -1.425 | 7.05E-06 | 1.62E-04 |
| miR-215-5p | -1.495 | 3.35E-06 | 9.15E-05 |
| miR-199a-5p | -1.543 | 4.41E-05 | 5.93E-04 |
| miR-342-5p | -1.561 | 2.57E-06 | 7.57E-05 |
| miR-224-5p | -1.572 | 3.86E-05 | 5.81E-04 |
| miR-126-3p | -1.580 | 2.91E-07 | 1.43E-05 |
| miR-381-3p | -1.592 | 1.62E-04 | 1.71E-03 |
| miR-495-3p | -1.598 | 1.91E-04 | 1.93E-03 |
| miR-142-3p | -1.743 | 2.79E-05 | 4.63E-04 |
| miR-11400 | -1.744 | 4.04E-05 | 5.84E-04 |
| miR-375-3p | -1.777 | 3.91E-07 | 1.79E-05 |
| miR-150-5p | -1.783 | 1.16E-05 | 2.37E-04 |
| miR-340-3p | -1.798 | 4.13E-07 | 1.79E-05 |
| miR-4433b-3p | -1.932 | 5.83E-04 | 4.94E-03 |

**Table E 3**: Comparison of healthy volunteers (baseline) to patients with severe COVID-19 ARDS. Significantly regulated miRNAs were derived from extracellular vesicles.

| **miRNA** | **Expr log ratio** | **Expr p-value** | **Expr False Discovery Rate (q-value)** |
| --- | --- | --- | --- |
| miR-542-3p | 8.323 | 2.92E-19 | 1.13E-16 |
| miR-1-3p | 6.239 | 1.98E-10 | 4.78E-09 |
| miR-378a-3p | 5.174 | 1.61E-17 | 4.14E-15 |
| miR-193a-5p | 5.115 | 3.00E-20 | 2.32E-17 |
| miR-338-5p | 4.788 | 1.15E-12 | 6.36E-11 |
| miR-502-3p | 4.497 | 6.64E-17 | 7.84E-15 |
| miR-450b-5p | 4.077 | 1.20E-11 | 4.42E-10 |
| miR-221-3p | 3.961 | 4.22E-13 | 2.71E-11 |
| miR-335-5p | 3.790 | 1.44E-08 | 2.52E-07 |
| miR-206 | 3.686 | 7.44E-04 | 3.38E-03 |
| miR-1246 | 3.386 | 2.92E-09 | 5.78E-08 |
| miR-20a-5p | 3.386 | 2.17E-11 | 7.28E-10 |
| miR-499a-5p | 3.227 | 7.50E-06 | 5.68E-05 |
| miR-501-3p | 2.782 | 4.66E-11 | 1.38E-09 |
| miR-23a-3p | 2.645 | 1.43E-07 | 1.84E-06 |
| miR-197-3p | 2.598 | 9.57E-08 | 1.30E-06 |
| miR-503-5p | 2.468 | 3.33E-06 | 2.92E-05 |
| miR-629-5p | 2.446 | 4.46E-12 | 1.91E-10 |
| miR-146a-5p | 2.427 | 1.01E-06 | 9.87E-06 |
| miR-361-5p | 2.417 | 2.68E-06 | 2.44E-05 |
| miR-576-3p | 2.381 | 9.62E-09 | 1.77E-07 |
| miR-223-5p | 2.345 | 2.19E-07 | 2.61E-06 |
| miR-93-5p | 2.331 | 9.47E-07 | 9.62E-06 |
| miR-152-3p | 2.308 | 1.04E-06 | 1.00E-05 |
| miR-30a-5p | 2.209 | 2.81E-06 | 2.53E-05 |
| miR-223-3p | 2.143 | 8.87E-04 | 3.94E-03 |
| miR-17-5p | 2.140 | 1.62E-04 | 9.01E-04 |
| miR-20b-5p | 2.108 | 2.79E-03 | 1.09E-02 |
| miR-143-3p | 2.040 | 1.41E-05 | 9.99E-05 |
| miR-30e-3p | -2.003 | 2.67E-05 | 1.81E-04 |
| miR-151a-3p | -2.036 | 2.95E-04 | 1.53E-03 |
| miR-425-5p | -2.142 | 1.65E-07 | 2.02E-06 |
| let-7a-5p | -2.183 | 1.63E-05 | 1.13E-04 |
| miR-144-3p | -2.202 | 1.78E-04 | 9.72E-04 |
| miR-1307-3p | -2.275 | 1.79E-04 | 9.72E-04 |
| miR-98-5p | -2.329 | 6.48E-07 | 6.94E-06 |
| miR-144-5p | -2.357 | 7.42E-05 | 4.66E-04 |
| miR-139-5p | -2.381 | 3.35E-06 | 2.92E-05 |
| miR-32-5p | -2.388 | 7.81E-04 | 3.52E-03 |
| miR-379-5p | -2.513 | 3.36E-03 | 1.27E-02 |
| miR-126-5p | -2.555 | 4.19E-07 | 4.75E-06 |
| let-7d-5p | -2.633 | 2.10E-08 | 3.45E-07 |
| miR-101-3p | -2.643 | 7.26E-06 | 5.55E-05 |
| miR-744-5p | -2.644 | 3.41E-12 | 1.63E-10 |
| let-7c-5p | -2.736 | 1.69E-07 | 2.04E-06 |
| miR-363-3p | -2.987 | 3.56E-06 | 2.99E-05 |
| miR-375-3p | -3.074 | 3.45E-06 | 2.96E-05 |
| miR-493-3p | -3.095 | 5.17E-04 | 2.45E-03 |
| miR-30c-5p | -3.165 | 3.58E-12 | 1.63E-10 |
| miR-150-5p | -3.170 | 3.55E-05 | 2.38E-04 |
| let-7b-5p | -3.363 | 1.71E-08 | 2.94E-07 |
| miR-432-5p | -3.841 | 2.11E-06 | 1.96E-05 |
| miR-26a-5p | -3.845 | 8.95E-15 | 7.67E-13 |
| miR-335-3p | -3.937 | 3.31E-07 | 3.87E-06 |
| miR-409-3p | -4.022 | 9.10E-07 | 9.37E-06 |
| miR-374a-5p | -4.075 | 1.65E-10 | 4.25E-09 |
| miR-126-3p | -4.090 | 4.20E-11 | 1.35E-09 |
| miR-495-3p | -4.198 | 1.24E-06 | 1.19E-05 |
| miR-215-5p | -4.203 | 1.16E-10 | 3.19E-09 |
| miR-381-3p | -4.251 | 7.50E-07 | 7.94E-06 |
| miR-224-5p | -4.365 | 2.55E-08 | 4.10E-07 |
| miR-543 | -4.708 | 4.17E-08 | 6.39E-07 |
| miR-370-3p | -4.777 | 4.48E-08 | 6.66E-07 |
| miR-342-5p | -5.061 | 2.01E-12 | 1.04E-10 |
| miR-142-3p | -5.520 | 3.19E-09 | 6.16E-08 |
| miR-4433b-5p | -5.802 | 1.45E-09 | 2.94E-08 |
| let-7e-5p | -6.083 | 1.53E-11 | 5.36E-10 |
| miR-654-3p | -6.734 | 1.76E-13 | 1.24E-11 |
| miR-11400 | -6.753 | 1.08E-10 | 3.08E-09 |
| miR-199a-5p | -7.653 | 1.14E-14 | 8.83E-13 |
| miR-340-3p | -7.965 | 6.58E-17 | 7.84E-15 |
| miR-4433b-3p | -23.617 | 9.71E-16 | 9.37E-14 |

**Table E 4:** Significantly regulated miRNAs derived from extracellular vesicles in patients with pneumonia (baseline) vs. patients with COVID-ARDS (Meidert et al., 2021).

| **miRNA** | **Expr log ratio** | **Expr p-value** | **Expr False Discovery Rate (q-value)** |
| --- | --- | --- | --- |
| miR-206 | 1.578150049 | 0.004864984 | 0.036045106 |
| miR-582-3p | 1.45950406 | 6.99E-05 | 0.002629597 |
| miR-1-3p | 1.437193473 | 0.000513645 | 0.008661113 |
| miR-200a-3p | 1.338348212 | 0.006201501 | 0.04222306 |
| miR-378a-3p | 1.203990761 | 1.44E-05 | 0.000607789 |
| miR-432-5p | -1.133790589 | 0.005827525 | 0.040709424 |
| miR-486-3p | -1.138234006 | 8.85E-05 | 0.002934423 |
| miR-340-3p | -1.195821546 | 0.000958821 | 0.012396257 |
| let-7a-5p | -1.203449681 | 3.81E-06 | 0.000230555 |
| let-7c-5p | -1.223557905 | 1.09E-05 | 0.000531066 |
| miR-335-3p | -1.283779539 | 0.000951173 | 0.012396257 |
| let-7d-5p | -1.32119613 | 1.09E-07 | 5.34E-05 |
| miR-654-3p | -1.36698854 | 0.000303854 | 0.006460193 |
| miR-199a-5p | -1.393018817 | 0.000318834 | 0.006496251 |
| let-7b-5p | -1.480364317 | 1.69E-06 | 0.000230555 |
| let-7e-5p | -1.80781685 | 3.15E-06 | 0.000230555 |
| miR-4433b-5p | -1.970879785 | 2.98E-06 | 0.000230555 |
| miR-3168 | -2.125953401 | 9.00E-05 | 0.002934423 |
| miR-1228-5p | -2.285965968 | 0.000285864 | 0.006353976 |
| miR-4433b-3p | -2,629750647 | 3,43E-06 | 0,000230555 |


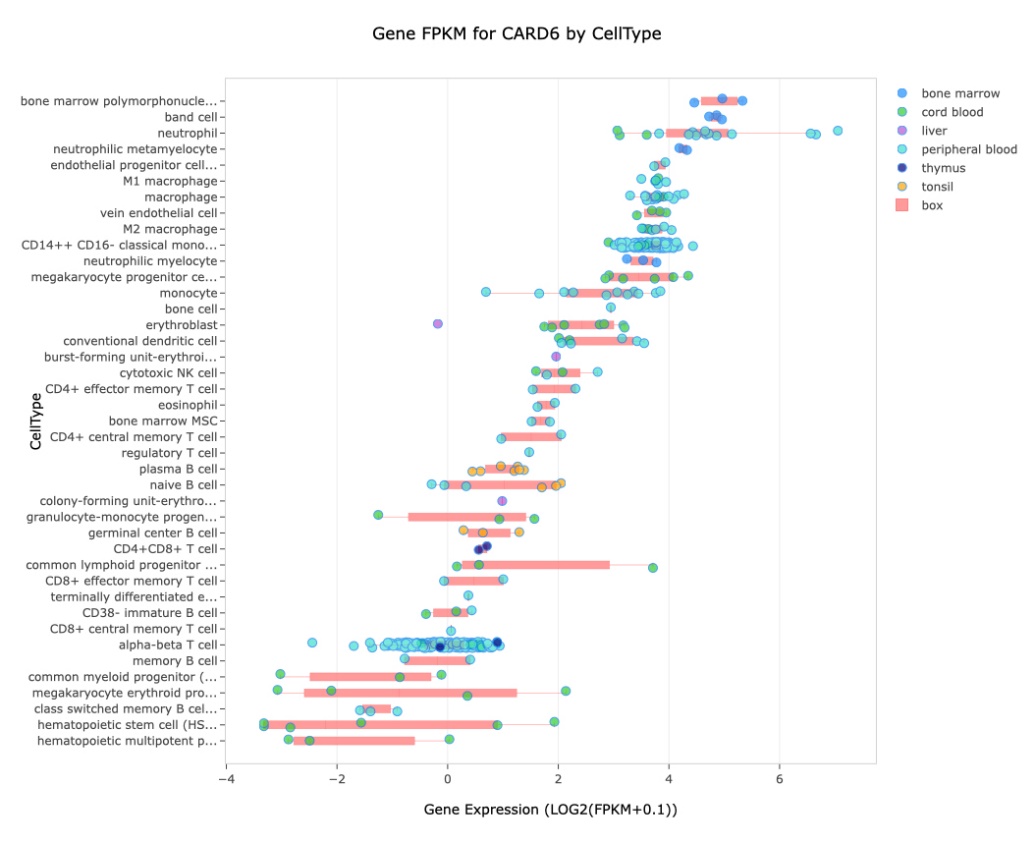


Figure e 2: Gene expression of CARD6 by cell type. Image from IPA BluePrint B38 GC33 (with permission).

**Reference**

Meidert, A.S., Hermann, S., Brandes, F., Kirchner, B., Buschmann, D., Billaud, J.-N., et al. (2021). Extracellular Vesicle Associated miRNAs Regulate Signaling Pathways Involved in COVID-19 Pneumonia and the Progression to Severe Acute Respiratory Corona Virus-2 Syndrome. Frontiers in Immunology 12(5287). doi: 10.3389/fimmu.2021.784028.
